# Nuclear localization of Toll/interleukin-1 receptor domain-containing adaptor protein reveals a previously unrecognized role in hepatic stellate cell activation

**DOI:** 10.3389/fimmu.2026.1870084

**Published:** 2026-08-21

**Authors:** Pramod Patidar, Rajat Atre, Mirza S. Baig

**Affiliations:** Mehta Family School of Biosciences and Biomedical Engineering, Indian Institute of Technology Indore (IITI), Indore, India

**Keywords:** alcohol, fibrosis, hepatic stellate cell (HSC), LPS, MyD88, TIRAP

## Abstract

**Background:**

The Toll/interleukin-1 receptor domain-containing adaptor protein (TIRAP) is a key adaptor in Toll-like receptor signaling, classically functioning as a cytoplasmic bridging adaptor for Myeloid differentiation primary response 88 (MyD88) recruitment. While its role in inflammatory signaling is well established, its contribution to alcohol-induced liver fibrosis remains unclear.

**Methods:**

We used lipopolysaccharide (LPS) and ethanol-stimulated hepatic stellate cells (LX-2) for *in vitro* analysis. Immunofluorescence and immunoblotting were performed to check TIRAP’s expression. siRNA was used for TIRAP silencing to monitor its significant activity. mRNA expression for pro-fibrotic markers was checked via qRT-PCR.

**Results:**

We demonstrated for the first time, nuclear translocation of TIRAP under alcoholic conditions. Confocal microscopy and nuclear–cytoplasmic fractionation confirmed TIRAP enrichment in the nucleus. Importantly, silencing of TIRAP in LX-2 significantly reduced the nuclear translocation and expression of fibrotic markers (such as *α-SMA* and *collagen*) in HSCs, suggesting a key role of nuclear TIRAP in driving fibrogenesis.

**Conclusion:**

Overall, these findings reveal a novel role for TIRAP beyond cytoplasmic signaling, suggesting potential nuclear functions that may regulate transcriptional programs driving inflammation and fibrosis.

## Introduction

The Toll-interleukin-1 receptor (TIR) domain-containing adaptor protein, also known as MyD88-adaptor-like (MAL), is a pivotal adaptor molecule in the innate immune system (1). Functionally, TIRAP acts as a bridging adaptor for several Toll-like receptors (TLR) family members, most notably TLR2 and TLR4, and facilitates downstream signaling by recruiting MyD88 to activated receptors. Through this mechanism, it governs the activation of key transcription factors such as nuclear factor κB (NF-κB) and activator protein 1 (AP-1), thereby triggering the expression of pro-inflammatory cytokines, chemokines, and other immune mediators essential for host defense (2). Since its discovery in 2001, TIRAP has been extensively studied and is now recognized as a major regulator of inflammatory signaling, largely owing to its ability to form complex protein–protein interaction networks through its conserved TIR domain (3). Dysregulation of TIRAP whether through altered expression, genetic polymorphisms, or functional modification has been implicated in a broad spectrum of disorders, including autoimmune conditions, chronic inflammatory diseases, microbial infections, and even tumorigenesis (4). In these pathological contexts, aberrant TIRAP-dependent signaling can exacerbate immune activation or impair the resolution of inflammation, underscoring its importance as both a homeostatic regulator and a potential therapeutic target. Traditionally, TIRAP has been described as a membrane-associated or cytoplasmic adaptor, functioning proximally to TLRs at the plasma membrane or endosomal compartments. Interestingly, a study has also suggested that TIRAP may interact with c-Jun, a subunit of the AP-1 transcription factor, particularly under conditions of LPS-dependent TLR4 activation (5). Beyond its classical role as an adaptor bridging MyD88 recruitment, emerging evidence suggests that TIRAP may directly modulate transcription factor activity. This introduces a novel layer of complexity to TIRAP-mediated signaling, positioning it as a key regulator that fine-tunes downstream inflammatory responses.

Chronic alcohol consumption is a primary driver of hepatic inflammation, oxidative stress, and stellate cell activation, which together lead to progressive liver fibrosis (6). Within the liver, injury-induced inflammatory cascades trigger resident Kupffer cells and infiltrating immune cells to release pro-inflammatory cytokines that stimulate hepatic stellate cells to transdifferentiate into collagen-producing myofibroblasts (7). TLR signaling, particularly through TLR4, plays a central role in driving this hepatic immune activation. As a necessary bridging adaptor that recruits MyD88 during TLR4 engagement, TIRAP represents a key node in governing these pro-inflammatory cascades (8). Mechanistically, alcohol exposure promotes hepatic pathology through dual, complementary mechanisms: chronic alcohol increases intestinal permeability, driving portal translocation of gut-derived LPS as a direct extracellular TLR4 agonist, while alcohol metabolites concurrently induce cellular stress and ROS that sensitize inflammatory pathways (9). While TIRAP has previously been implicated as a mediator in radiation-induced liver injury (10), its specific contribution to alcoholic liver pathology remains incompletely understood. These prompted us to examine whether TIRAP functions as a critical signaling adaptor influencing transcriptional programs that drive inflammation and fibrosis in alcohol-related liver disease.

## Methods

### Cell culture

The LX-2 cell line was maintained in Dulbecco’s Modified Eagle Medium (DMEM, Gibco ThermoFisher Scientific India) supplemented with 10% FBS (Gibco, ThermoFisher Scientific India) and 1% penicillin/streptomycin in an incubator at 37 °C with 5% CO2. To mimic the alcoholic liver disease, LX-2 cells were treated with LPS (1 μg/ml) and ethanol (25 mM) together for 24hrs.

### siRNA transfection and TIRAP silencing

LX-2 cells were seeded in 6-well plates at a density of 0.5 × 10^5^ cells per well, and upon reaching 80% confluency, the cells were transfected with TIRAP-specific siRNA (Santa Cruz Cat. No.- sc-42932) at concentrations of 50 nM. For transfection, TIRAP siRNA was diluted in 100 µL of serum-free media, and separately, 5 µL of Lipofectamine transfection reagent was diluted in 100 µL of serum-free media. Both solutions were combined and incubated at room temperature for 45 minutes to allow complex formation. The resulting siRNA-lipid complexes were then added to macrophages in wells containing 800 µL of serum-free media, and the cells were incubated with the complexes for 4–6 hours. Following this, 1 mL of complete medium supplemented with 2X serum concentration was added to each well, and incubation continued for an additional 24 hours.

### Immunofluorescence

LX-2 cells were seeded on coverslips in 12-well plates (0.5 × 10^5^ cells/well). For fluorescence staining, after treatment, cells were washed with PBS and fixed in 4% paraformaldehyde for 15 min and permeabilized with 0.1% Triton-X 100 for 10 min. Then, the cells were incubated with 5% blocking reagent at room temperature to block nonspecific binding sites. After that, cells were incubated with primary antibodies against TIRAP (Santa Cruz, USA), p-TIRAP (Invitrogen, USA) overnight at 4 °C, followed by incubation with FITC-conjugated Alexa Fluor 488 and Alexa Fluor 594. Nuclear counterstaining was performed using DAPI mounting medium (Thermo Fisher Scientific, USA) according to the manufacturer’s instructions. Stained cells were analyzed using an Olympus confocal laser scanning microscope.

### Immunoblotting

Cells were collected and washed three times with phosphate-buffered saline (PBS) before being lysed in radioimmunoprecipitation assay (RIPA) buffer supplemented with protease and phosphatase inhibitors. Total protein concentration was quantified using the Bradford protein assay (Bio-Rad, USA). Equal amounts of protein were then resolved by SDS-PAGE and transferred onto nitrocellulose membranes (Bio-Rad, USA). The membranes were blocked with 5% BSA (HiMedia, India) for 1 hour at room temperature and subsequently incubated with the appropriate primary antibodies overnight at 4 °C. Following primary antibody incubation, the membranes were washed three times with TBST and then incubated with horseradish peroxidase (HRP)–conjugated secondary antibodies for 1 hour at room temperature. The blots were then incubated with the chemiluminescence HRP Substrate (Bio-Rad, USA) and imaged using a gel doc (Image Quant LAS 4000, GE Healthcare, Uppsala, Sweden) following a second TBST wash. ImageJ (National Institutes of Health; doi.org/10.1038/nmeth.2089) was used to determine band intensities. The antibodies used were as follows: TIRAP (Santa Cruz, USA), p-TIRAP (Invitrogen, USA), β-Actin (Invitrogen, USA), & secondary HRP conjugated antibody.

### Nuclear cytoplasmic assay

The LX-2 cells were washed with PBS, and Cytoplasmic and nuclear proteins were isolated using the Nuclear/Cytosol Fractionation Kit (BioVision, USA, Cat. No. K266-100) according to the manufacturer’s instructions. Briefly, cells were harvested by centrifugation (600×g, 5 min, 4 °C), re-pelleted (500×g, 2–3 min), and resuspended in 0.2 mL Cytosol Extraction Buffer A (CEB-A) mix. After a 10-minute incubation on ice, 11 μl of ice-cold CEB-B was added. The lysate was vortexed, incubated on ice for 1 minute, and centrifuged at 16,000×g for 5 minutes at 4 °C to yield the cytoplasmic fraction (supernatant). The nuclear pellet was resuspended in 100 μl ice-cold Nuclear Extraction Buffer (NEB) mix and incubated on ice for 40 minutes with 15-second vertexing every 10 minutes. Following centrifugation (16,000×g, 10 min, 4 °C), the cytoplasmic and nuclear extract was further used for western blot analysis. Fraction purity and protein loading were validated via Western blot analysis using β-actin as a cytoplasmic marker and HDAC1 as a nuclear marker.

### Quantitative real-time polymerase chain reaction

A qRT-PCR analysis was performed to investigate the expression of fibrotic markers including *α-SMA*, and *Collagen* (colo1A1) in LX-2 cells, using the standardized procedures described earlier. Following the treatment, cell pellets were collected, and RNA isolation was carried out using TRIZol reagent (Takara, Shiga, Japan). Subsequently, cDNA synthesis was conducted using PrimeScript RT Master Mix (Takara, Shiga, Japan). The expression levels of fibrotic markers in LX-2 cells were quantified via qRT-PCR with SYBR green (Applied Biosystems, Waltham, USA) and gene-specific primers (**Supplementary Table 2**) on Stepone (Thermo Fisher Scientific). The gene expression level was normalized to that of GAPDH.

### Statistical analysis

The experimental outcomes were executed three independent biological replicates. A t-test and one-way ANOVA followed by Tukey’s multiple comparison *post-hoc* test was implemented in the statistical evaluation of the data. P values were estimated using GraphPad Prism version 8, and p values of *0.01, 0.001*, and *0.0001* were considered statistically significant and represented by *, **, and ***, denoting upregulation/downregulation, respectively.

## Results

### Ethanol and LPS induce nuclear translocation of TIRAP in hepatic stellate cells

**Figure 1:**
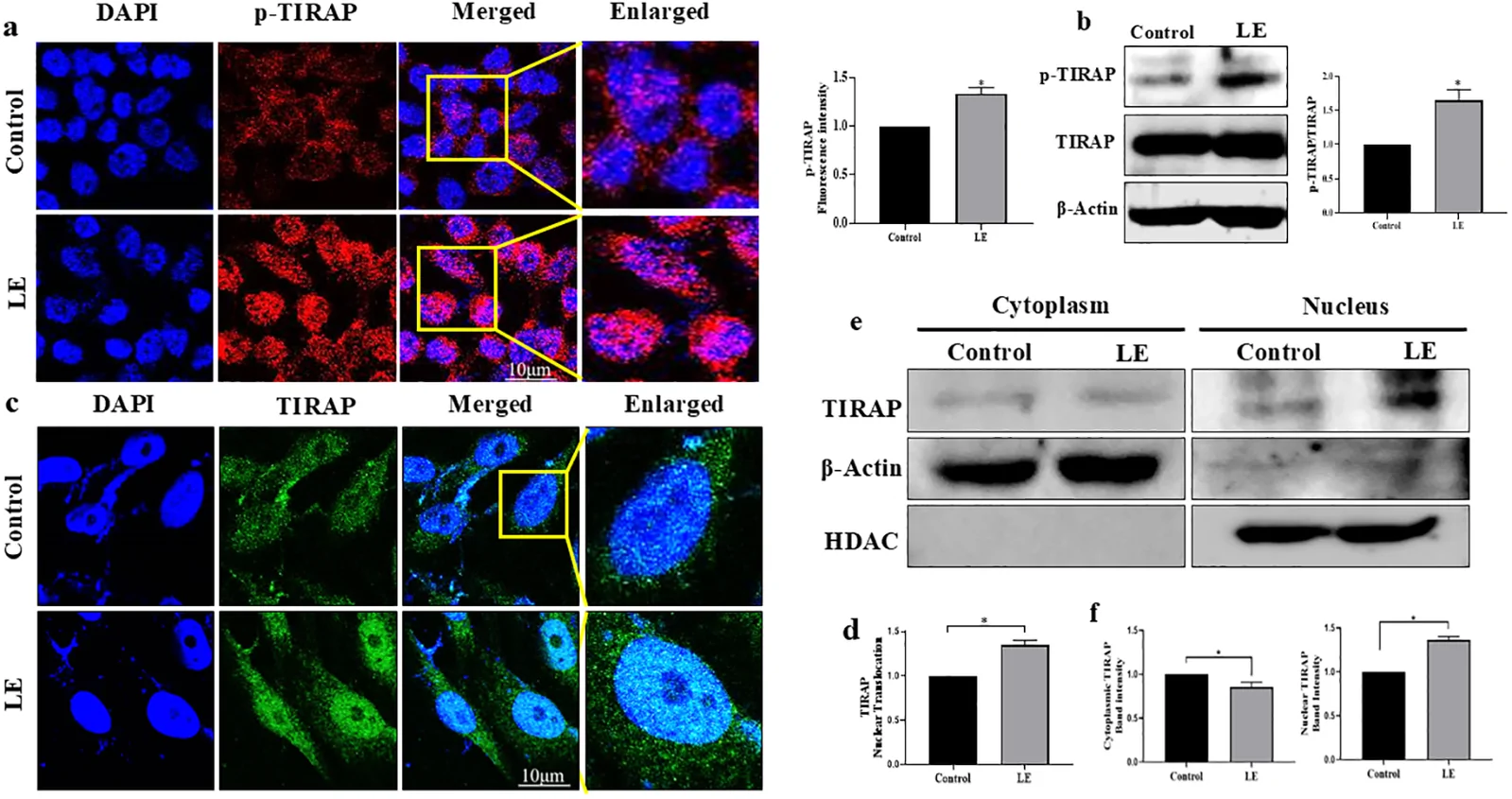
Ethanol and LPS (LE) stimulate TIRAP phosphorylation and nuclear translocation in HSCs. **(a)** Confocal immunofluorescence microscopy showing increased cellular phosphorylation of TIRAP in HSCs exposed to ethanol (25mM) and LPS (1μg/ml) (LE) for 24 h. TIRAP phosphorylation was detected using primary antibodies against phospho-TIRAP and Alexa Fluor 594-tagged (red) secondary antibodies. Scale bar = 10μm. **(b)** Western blot analysis demonstrates enhanced cellular phosphorylation of TIRAP upon ethanol and LPS treatment, probed with specific primary antibodies for total TIRAP and phospho-TIRAP alongside secondary antibodies. **(c, d)** Confocal immunofluorescence images showing nuclear translocation and accumulation of TIRAP in HSCs following treatment. Total TIRAP was detected using specific primary antibodies for TIRAP and Alexa Fluor 488-tagged (green) secondary antibodies. Scale bar = 10 μm. **(e, f)** Nuclear and cytoplasmic fractionation assay confirming the enrichment of TIRAP in the nuclear fraction following ethanol and LPS exposure. Data are representative of three independent experiments and are presented as mean ± SD. Statistical analysis was performed using a one-way ANOVA (*P =0.01,**P=0.001, ***P=0.0001, ****P = 0.00001).

To investigate whether TIRAP activation contributes to inflammation and fibrosis in alcohol-related liver disease, we employed LX-2 hepatic stellate cells as an experimental model. Initially, we examined whether ethanol exposure induces phosphorylation of TIRAP. LX-2 cells were treated with 25 mM ethanol for 24 hours and analyzed using a phospho-TIRAP–specific antibody (**Figures 1a, b**). As anticipated, we found ethanol-dependent increase in TIRAP phosphorylation (activation) via both immunofluorescence as well as immunoblotting. Interestingly, at the cytosol or plasma membrane, immunocytochemical analysis revealed that the activation was found to be accompanied with pronounced nuclear localization of TIRAP. Distinct punctuated nuclear staining was observed, clearly contrasting with its commonly described cytoplasmic distribution. This unexpected finding was further validated using nuclear–cytoplasmic fractionation assays, which demonstrated consistent enrichment of TIRAP in nuclear fractions accompanied by reduced levels in the cytoplasmic compartment (**Figures 1c–f**). Notably, this shuttling was strictly condition- and cell-type-specific, as nuclear entry was not observed under single-treatment conditions or in non-hepatic immune cells (**Supplementary Figure 5**). Additionally, combined treatment with LPS and ethanol further enhanced nuclear localization of TIRAP, mimicking alcoholic liver disease conditions and confirming that this translocation is a reproducible and biologically regulated event.

### TIRAP knockdown reduces nuclear localization and suppresses fibrogenic marker expression

**Figure 2:**
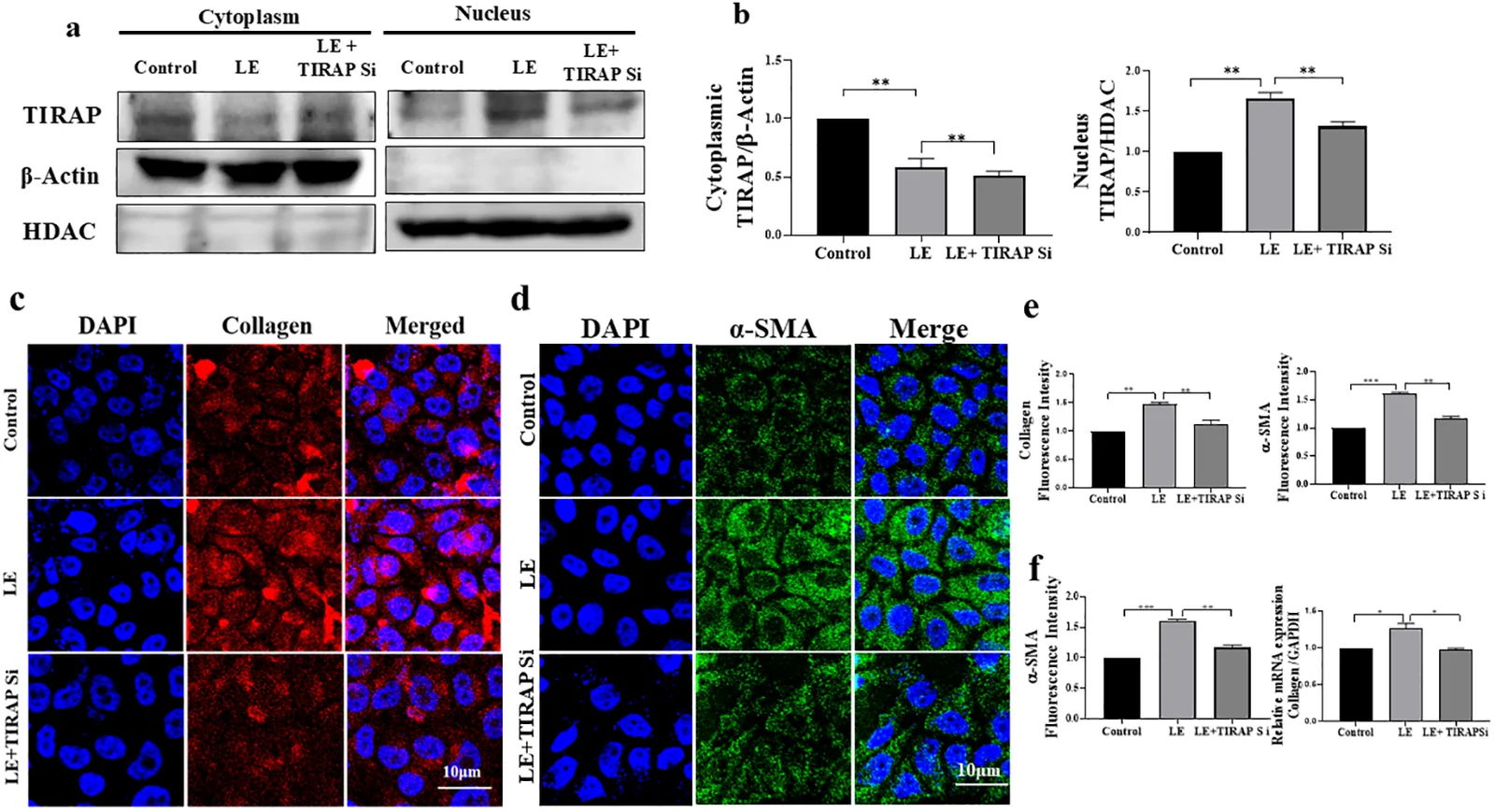
TIRAP knockdown attenuates ethanol- and LPS-induced activation and fibrotic responses in hepatic stellate cells (HSCs). **(a, b)** Nuclear and cytoplasmic fractionation assay demonstrating the suppression of TIRAP expression and nuclear translocation under TIRAP silencing conditions in HSCs exposed to ethanol (25 mM) and LPS (1 μg/ml) for 24 hours. **(c, d)** Confocal immunofluorescence images showing reduced cellular expression of collagen and α-SMA upon TIRAP knockdown in ethanol and LPS treated HSCs. Collagen expression was probed using specific primary antibodies and Alexa Fluor 594-tagged (red) secondary antibodies **(c)** α-SMA expression was probed using specific primary antibodies and Alexa Fluor 488-tagged (green) secondary antibodies **(d)**. Scale bar = 10μM. **(e)** RT-PCR analysis revealing decreased mRNA expression levels of α-SMA and collagen in TIRAP-silenced HSCs following ethanol and LPS exposure. Data are representative of three independent experiments and are presented as mean ± SD. Statistical analysis was performed using a one-way ANOVA (*P =0.01,**P=0.001, ***P=0.0001, ****P = 0.00001).

To confirm the requirement of TIRAP for nuclear translocation under alcoholic conditions, TIRAP expression was silenced in LX-2 cells using siRNA. Knockdown of TIRAP markedly diminished its expression and nuclear localization following ethanol and LPS exposure (**Figures 2a, b**). Next, to determine the functional relevance of nuclear TIRAP in fibrogenesis, we examined the expression of key extracellular matrix components and fibrotic markers. Confocal microscopy revealed that silencing TIRAP significantly reduced levels of α-SMA and collagen compared with control cells (**Figures 2c–e**). The observed reduction of α-SMA and collagen at protein level via confocal microscopy was further confirmed at mRNA level via RT-PCR where decreased expression *α-Sma* and *Collagen* was found that aligned with previous results (**Figure 2f**). These findings suggest that nuclear TIRAP plays a critical role in activating fibrogenic signaling pathways in hepatic stellate cells.

## Discussion

Liver fibrosis is the end stage of most chronic liver diseases. Liver fibrosis and hepatic stellate cell activation may result from bacterial and viral infections, and chronic alcohol consumption (11). It is a progressive and multistage process which involves the interaction of various parenchymal and non-parenchymal cells, such as tissue resident and circulating macrophage, liver endothelial cells, hepatocytes and hepatic stellate cells (12). In the context of alcoholic liver disease (ALD), chronic alcohol consumption impairs the function of the gut barrier and increases gut permeability, leading to an imbalance in gut microbiota populations. This facilitates increased blood serum endotoxin (LPS) level (13).

To initiate the downstream response, endotoxin LPS acts as a potent activator of the TLR4 receptor which is widely expressed throughout the liver, including in both parenchymal and non-parenchymal cells (14, 15). Activation of TLR4 initiates a series of signaling pathways that ultimately lead to the production of inflammatory cytokines (15, 16). Furthermore, TLR4 stimulation recruits cytoplasmic and membrane-bound adaptor proteins such as TIRAP and MyD88, facilitating activation of transcription factors and innate immune responses driving HSC fibrogenesis (17, 18). TIRAP is a key upstream inflammatory adaptor protein that participates in the majority of the TLR-mediated signaling events (19). It is known to engage in a variety of protein-protein interactions, playing a crucial role in the complex signaling events that underline different inflammatory conditions (5, 20). While the involvement of TLR4-driven inflammatory signaling in alcoholic liver disease (ALD) is well established, much less attention has been given to the role of adaptor proteins that fine-tune these pathways. In particular, the contribution of TIRAP in alcohol-induced liver inflammation remains largely unexplored. Most previous studies have emphasized receptor activation or downstream transcriptional responses, leaving adaptor-level regulation insufficiently understood.

To investigate the involvement of TIRAP in driving intracellular signaling during alcoholic liver fibrosis, we first assessed TIRAP-mediated signaling in LX-2 cells. Immunofluorescence and immunoblotting studies of TIRAP in LX-2 cells revealed that exposure to LE significantly activated TIRAP by inducing its phosphorylation (**Figures 1a, b**). In support of this, Chen et al. have also suggested that in HCC, radiation induces TIRAP-mediated HSCs activation (10). Furthermore, analysis of a public transcriptomic dataset (GSE155907) consistently showed elevated *TIRAP* gene expression in alcoholic samples compared to healthy controls (**Supplementary Figure 1**).

Following its activation, downstream signaling is mediated through subcellular localization changes. Subsequent investigations via immunofluorescence and nuclear-cytoplasmic assay suggest that under alcoholic conditions, TIRAP is not only confined to the cytoplasm but also translocate to the nucleus (**Figures 1c–f**). These results imply that TIRAP regulates HSCs activation in the cytoplasm through downstream signaling pathways involved in fibrogenic responses. In addition, our results indicate TIRAP translocation in the nucleus, suggesting TIRAP might be involved in nuclear signaling governing HSCs activation, and ECM production (**Figures 2c–f**). To confirm the functional link between TIRAP and fibrogenic gene activation, silencing of TIRAP in LX-2 cells showed reduced expression of -SMA and Collagen, indicating the direct involvement of TIRAP in the regulation of genes associated with fibrosis progression. This aligns with recent findings by Zhang et al. (2022), which demonstrated that TLR adaptor signaling within hepatic stellate cells directly regulates pro-inflammatory crosstalk and exacerbates fibrotic progression (21).

Our findings indicate that TIRAP functions as an important signaling node, which is in line with previous studies reported by Rajpoot et al. that highlight TIRAP’s interaction with multiple proteins, thereby influencing key downstream pathways that drive hepatic inflammation (3). The observed alterations in TIRAP signaling under alcohol-induced conditions suggest that this adaptor plays a previously unrecognized role in shaping inflammatory outcomes in ALD.

**Figure 3:**
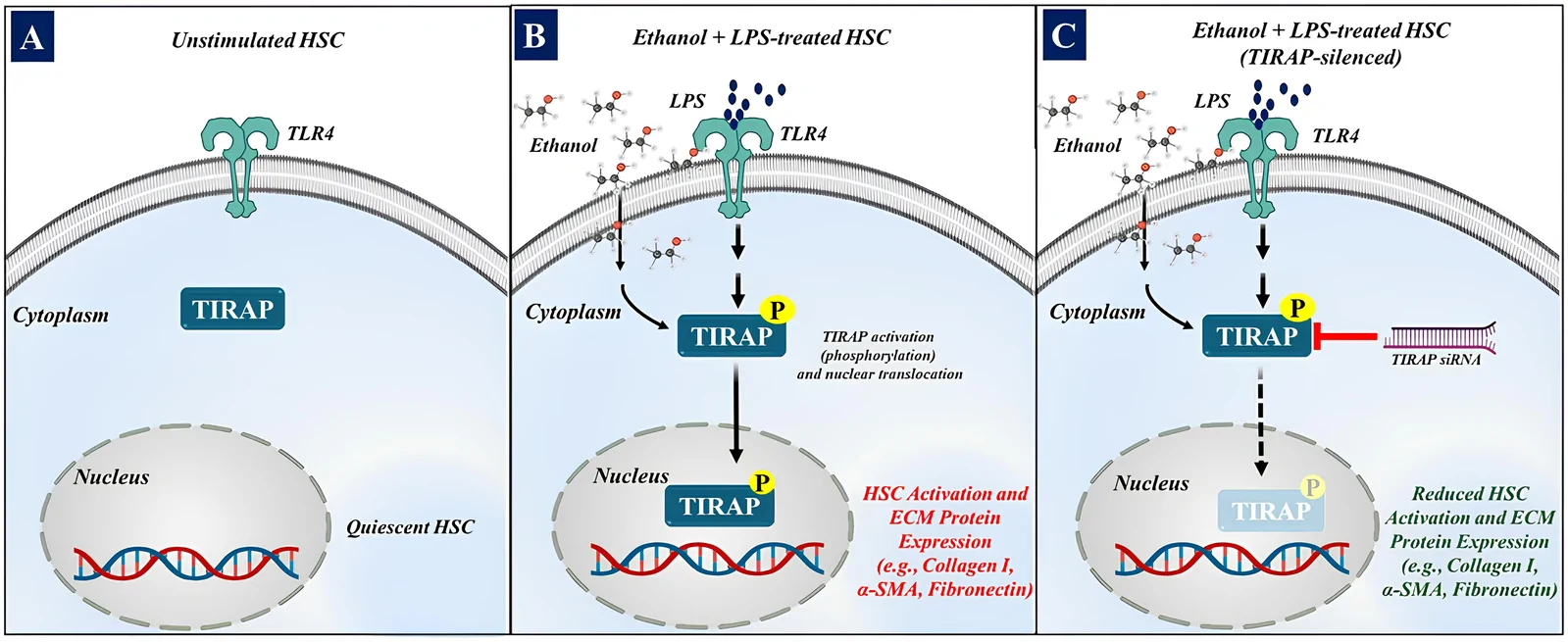
Proposed mechanism of TIRAP-mediated signalling in response to ethanol and LPS and the effect of TIRAP knockdown. **(A)** Under basal conditions, TIRAP remains predominantly inactive in the cytoplasm, with minimal nuclear localization and limited HSC activation and ECM protein expression. **(B)** Combined exposure to ethanol and lipopolysaccharide (LPS) promotes TIRAP phosphorylation and activation in HSCs, facilitating its nuclear translocation and promoting transcriptional activity associated with ECM protein expression and HSC activation. **(C)** Conversely, TIRAP knockdown using siRNA attenuates TIRAP activation and nuclear translocation in response to ethanol and LPS, resulting in reduced ECM protein expression and suppression of HSC activation.

Collectively, these results trace a clear pathway from upstream TLR4 activation to intracellular TIRAP translocation, ultimately driving HSC activation and fibrotic progression in alcohol-induced liver injury (**Figure 3**). Traditionally viewed as a cytoplasmic adaptor mediating Toll-like receptor signaling, TIRAP now emerges as a potential modulator of nuclear processes, possibly through interactions with transcription factors such as c-Jun, which are known to regulate fibrogenic gene expression (5). In support of this nuclear capacity, *in silico* sequence and disorder profiling revealed a functional N-terminal bipartite nuclear localization signal (NLS) embedded within an intrinsically disordered region (residues 1–78) of TIRAP (**Supplementary Figures 1**–**4**). This structural flexibility likely facilitates signaling-induced conformational changes required for nuclear import and chromatin-associated interactions. By amplifying pro-inflammatory and pro-fibrotic signals from within the nucleus, nuclear TIRAP may represent a key checkpoint in alcoholic liver fibrosis. Overall, establishing TIRAP as a critical player in hepatic stellate cell activation opens new avenues for understanding the molecular underpinnings of liver fibrosis. Targeting adaptor-level signaling nodes like TIRAP may offer promising therapeutic strategies to disrupt chronic inflammatory and fibrotic cascades in liver disease. Future research focusing on the broader interactome and downstream signaling networks of TIRAP will be essential to translate these mechanistic insights into effective targeted therapies.

## Conclusion

The study provides the first direct evidence of TIRAP translocating to the nucleus in a model of liver fibrosis, revealing a novel and previously unrecognized aspect of TIRAP biology. This nuclear localization suggests that TIRAP may have functions beyond its established role in cytoplasmic signaling, potentially influencing gene expression, chromatin dynamics, or other nuclear processes that contribute to fibrogenesis. The identification of nuclear TIRAP opens important questions regarding its regulatory mechanisms, interaction partners, and downstream effects in liver fibrosis. Future studies aimed at mapping TIRAP’s nuclear interactome and dissecting its functional consequences will be critical to determining whether TIRAP acts as a mechanistic driver of liver pathology. Ultimately, these insights could position TIRAP as a potential therapeutic target, paving the way for novel strategies to treat liver fibrosis and associated malignancies.

## Data Availability

The original contributions presented in this study are included in the article and **Supplementary Material**. Further inquiries can be directed to the corresponding author(s).

## References

[1] Fitzgerald KA Palsson-McDermott EM Bowie AG Jefferies CA Mansell AS Brady G . Mal (MyD88-adapter-like) is required for Toll-like receptor-4 signal transduction. Nature. (2001) 413:78–83. doi: 10.1038/35092578 11544529

[2] Bernard NJ O’Neill LA . Mal, more than a bridge to MyD88. IUBMB Life. (2013) 65:777–86. doi: 10.1002/iub.1201 23983209

[3] Rajpoot S Wary KK Ibbott R Liu D Saqib U Thurston TLM . TIRAP in the mechanism of inflammation. Front Immunol. (2021) 12:697588. doi: 10.3389/fimmu.2021.697588 34305934 PMC8297548

[4] Belhaouane I Hoffmann E Chamaillard M Brodin P Machelart A . Paradoxical roles of the MAL/Tirap adaptor in pathologies. Front Immunol. (2020) 11:569127. doi: 10.3389/fimmu.2020.569127 33072109 PMC7544743

[5] Srivastava M Saqib U Banerjee S Wary K Kizil B Muthu K . Inhibition of the TIRAP-c-Jun interaction as a therapeutic strategy for AP1-mediated inflammatory responses. Int Immunopharmacol. (2019) 71:188–97. doi: 10.1016/j.intimp.2019.03.031 30909134

[6] Ceni E Mello T Galli A . Pathogenesis of alcoholic liver disease: Role of oxidative metabolism. WJG. (2014) 20:17756–72. doi: 10.3748/wjg.v20.i47.17756 25548474 PMC4273126

[7] Seki E Schwabe RF . Hepatic inflammation and fibrosis: Functional links and key pathways. Hepatology. (2015) 61:1066–79. doi: 10.1002/hep.27332 25066777 PMC4306641

[8] Soares JB Pimentel-Nunes P Roncon-Albuquerque R Leite-Moreira A . The role of lipopolysaccharide/toll-like receptor 4 signaling in chronic liver diseases. Hepatol Int. (2010) 4:659–72. doi: 10.1007/s12072-010-9219-x 21286336 PMC2994611

[9] Pabst O Hornef MW Schaap FG Cerovic V Clavel T Bruns T . Gut–liver axis: barriers and functional circuits. Nat Rev Gastroenterol Hepatol. (2023) 20:447–61. doi: 10.1038/s41575-023-00771-6 37085614

[10] Chen Y Zhou P Deng Y Cai X Sun M Sun Y . ALKBH5‐mediated m^6^ A demethylation of TIRAP mRNA promotes radiation‐induced liver fibrosis and decreases radiosensitivity of hepatocellular carcinoma. Clin Trans Med. (2023) 13:e1198. doi: 10.1002/ctm2.1198 36792369 PMC9931500

[11] Poynard T Mathurin P Lai CL Guyader D Poupon R Tainturier MH . A comparison of fibrosis progression in chronic liver diseases. J Hepatol. (2003) 38:257–65. doi: 10.1016/S0168-8278(02)00413-0 12586290

[12] Berumen J Baglieri J Kisseleva T Mekeel K . Liver fibrosis: Pathophysiology and clinical implications. WIREs Mech Dis. (2021) 13:e1499. doi: 10.1002/wsbm.1499 32713091 PMC9479486

[13] Parlesak A Schäfer C Schütz T Bode JC Bode C . Increased intestinal permeability to macromolecules and endotoxemia in patients with chronic alcohol abuse in different stages of alcohol-induced liver disease. J Hepatol. (2000) 32:742–7. doi: 10.1016/S0168-8278(00)80242-1 10845660

[14] Park BS Lee JO . Recognition of lipopolysaccharide pattern by TLR4 complexes. Exp Mol Med. (2013) 45:e66. doi: 10.1038/emm.2013.97 24310172 PMC3880462

[15] Seki E Park E Fujimoto J . Toll‐like receptor signaling in liver regeneration, fibrosis and carcinogenesis. Hepatol Res. (2011) 41:597–610. doi: 10.1111/j.1872-034X.2011.00822.x 21696522 PMC3754784

[16] Szabo G Dolganiuc A Dai Q Pruett SB . TLR4, Ethanol, and Lipid Rafts: A new mechanism of ethanol action with implications for other receptor-mediated effects. J Immunol. (2007) 178:1243–9. doi: 10.4049/jimmunol.178.3.1243 17237368

[17] Thapa M Chinnadurai R Velazquez VM Tedesco D Elrod E Han J . Liver fibrosis occurs through dysregulation of MyD88‐dependent innate B‐cell activity. Hepatology. (2015) 61:2067–79. doi: 10.1002/hep.27761 25711908 PMC4441566

[18] Kagan JC Medzhitov R . Phosphoinositide-mediated adaptor recruitment controls Toll-like receptor signaling. Cell. (2006) 125:943–55. doi: 10.1016/j.cell.2006.03.047 16751103

[19] Sakaguchi M Murata H Yamamoto K Ono T Sakaguchi Y Motoyama A . TIRAP, an adaptor protein for TLR2/4, transduces a signal from RAGE phosphorylated upon ligand binding. PloS One. (2011) 6:e23132. doi: 10.1371/journal.pone.0023132 21829704 PMC3148248

[20] Rajpoot S Kumar A Zhang KYJ Gan SH Baig MS . TIRAP-mediated activation of p38 MAPK in inflammatory signaling. Sci Rep. (2022) 12:5601. doi: 10.1038/s41598-022-09528-8 35379857 PMC8979995

[21] Zhang J Liu Y Chen H Yuan Q Wang J Niu M . MyD88 in hepatic stellate cells enhances liver fibrosis via promoting macrophage M1 polarization. Cell Death Dis. (2022) 13:411. doi: 10.1038/s41419-022-04802-z 35484116 PMC9051099

